# Michelangelo Effect in Virtual Sculpturing: Prospective for Motor Neurorehabilitation in the Metaverse

**DOI:** 10.5334/joc.345

**Published:** 2024-01-29

**Authors:** Simona Pascucci, Giorgia Forte, Elena Angelini, Franco Marinozzi, Fabiano Bini, Gabriella Antonucci, Marco Iosa, Gaetano Tieri

**Affiliations:** 1 Department of Mechanical and Aerospace Engineering, Sapienza University of Rome, Rome, Italy; 2 IRCCS Santa Lucia Foundation, Rome, Italy; 3 Department of Psychology, Sapienza University of Rome, Rome, Italy; 4 Virtual Reality and Digital Neuroscience Lab, Department of Law and Digital Society, University of Rome UnitelmaSapienza, Rome, Italy

**Keywords:** Immersive Virtual Reality, motor rehabilitation, Art therapy, Kinematics, Metaverse

## Abstract

We investigated the Michelangelo effect, i.e. the facilitatory effect of a virtual art therapy in motor rehabilitation (Iosa et al. 2021), with a novel virtual reality paradigm in which users are engaged in motor exercises with 3D sculptures. In particular, thirty young adults were immersed in a virtual environment where they could sculpt, by using the real hands, some famous sculptures in the history of art, such as the David of Michelangelo, the Venus of Milo and the statue of Laocoon and His Sons, and their control stimuli, i.e. statues in very low resolution or cubes. We recorded the kinematics (length, the time to complete each trial, mean normalized jerk) and questionnaire answers (objective and subjective beauty, User Satisfaction Evaluation Questionnaire and Nasa Task Load Index). In general, we found that the perception of subjective and objective beauty was higher when sculpting the statues than control stimuli, the judgment of usability of the system was high. The perceived fatigue was not higher when sculpting the statues despite the longer time spent in completing the task that with respect to the control stimuli. Moreover, we found that the interaction with the experimental statues affected the fluidity and symmetry of hands movements. Finally, we discuss this evidence regarding the art therapy and neuroaesthetics principles for motor rehabilitation in the Metaverse with VR, including the possible role of virtual embodiment (illusory feeling to have a virtual body) for boosting the efficacy of the clinical applications.

## 1. Introduction

Recent advances in computer technology, such as the development of new generations of virtual reality (VR) head mounted displays (HMDs) and software for 3D modelling and VR programming, have opened new avenues for studying the human brain and behaviour and for realizing novel clinical applications (Tieri et al. 2018; Fusco & Tieri, 2022). Indeed, evidence suggests that the exposure to an immersive VR elicits (a) the illusory sensation of ‘being physically present’ in the virtual environment, i.e. sense of presence (Sanchez-Vives and Slater, 2005), which allows one to respond in a realistic way to the virtual stimuli and elicits physiological reactions “as if” the subject is physically situated in a real place (Riva et al. 2007; Meehan et al. 2002), and (b) the illusory sensation to ‘wear another body’, by observing and controlling a virtual body from a first person perspective point of view, this is called the embodiment illusion (Kilteni et al. 2012; Tieri et al. 2015a, 2017). Moreover, VR technology offers more ecological validity and allows people to interact in a naturalistic way with virtual stimuli by using real body movements for reaching and grasping stimuli or walking and navigating inside the virtual environments (Bohil et al. 2011). Recently, it has been proposed a conceptualization of the Metaverse, i.e. a social virtual environment where users can enter inside, wear the own virtual avatar and feel a “deep feeling of presence” through a multi-sensory experience (Riva et al., 2021; Riva & Wiederhold 2022). The Metaverse could potentially revolutionize our communications and social connections in near future and importantly, could be promising for realizing novel clinical approaches, where medical doctors and patients, even if physically dislocated in different area, can enter together in the same virtual space. However, despite the efforts done so far (Riva et al., 2023), the evidence is still few and more investigations are needed in this regards. Recently, novel results coming from neuroaesthetics field, which highlight the efficacy of virtual art therapy for motor rehabilitation, can be a good and smart solution for expand the clinical application in VR and, in general, also in the Metaverse.

The use of VR in neurorehabilitation offers the advantages of simulating a computer-generated environment that closely resembles reality, providing highly detailed situations that can be too dangerous, expensive, or impossible to recreate in physical reality, under the full control of the researcher or therapist (Tieri et al., 2018). VR can also enhance patient compliance and even provide amusement compared to the sometimes tedious conventional rehabilitation sessions. It can enable the recording of quantitative data on patient kinematic performances and introduce the possibility of tele-rehabilitation between patients and therapists within the Metaverse (Calabrò et al., 2022). In the follow, we introduce recent results in this direction, in particular regarding the ‘Michelangelo effect, i.e. the facilitatory effect of a virtual art therapy in motor rehabilitation obtained by painting a virtual masterpiece from art history (Iosa et al. 2021), and discuss how neuroaesthetics principles can be implemented in the Metaverse for increasing clinical efficacy in motor rehabilitation.

The work of the famous Italian artist Michelangelo inspired many different fields of humanities, not only art, but also including psychology and neuroscience. His statement “every block of stone has a statue inside it and it is the task of the sculptor to discover it” inspired the development of a protocol of art therapy for the neurorehabilitation of patients with stroke using virtual reality (Iosa et al., 2021). The authors referred to the observed reduction in fatigue and improvement in performance in artistic painting compared to the control condition as the Michelangelo effect [not to be confused with the “Michelangelo Phenomenon” defined by psychologist as the capacity of a person to positively modify the partner (Drigotas et al., 1999)].

The World Health Organization reported that art may increase health and wellness, thanks to aesthetic engagement, powerful involvement of imagination, sensory activation, evocation of emotions, cognitive stimulation, and in some cases favouring physical activity and social interaction (Fancourt and Finn, 2019). Psychological, physiological, social and behavioural responses may be evoked by art thanks to receptive engagement (such as watching a famous painting or statue) or active engagement (such as painting or sculpting) (Fancourt and Finn, 2019). The mere observation of an artwork has been found associated with imagination, aesthetic engagement, sensory activation, feelings of pleasure, cognitive stimulation, and emotional evocation. These responses are attributed to a broad and relatively spontaneous brain arousal in the viewer, brought about by the activation of dopaminergic neural networks and even responses in motor areas (Oliva et al., 2023). VR can merge receptive and active engagement, giving participants the illusion of being able to recreate an artistic masterpiece. In previous studies, we developed a novel system where participants sat wearing an HMD, and held, in a hand, a joystick, which allowed to interact with the virtual stimuli. The virtual environment consisted of a large and comfortable room, in the middle of which there was a canvas on an easel. The subject could interact with the canvas with a spherical brush, displayed in VR in the same place as the participant’s real hand. Each virtual canvas appeared white at the beginning of the task. Participants were instructed that the brush could color the canvas when put in contact with it, forming a painting. When the participant touched the virtual panel, the target white pixels were automatically deleted, revealing a part of the underlying historical artistic masterpiece, creating the illusion of painting (Iosa et al., 2021, 2022; De Giorgi et al., 2023). This VR protocol combines the active motor task of painting with the cited engagement of observing of an artistic masterpiece. Participants perceived less fatigue when they had the illusion of recreating an art masterpiece compared to the control condition in which they simply coloured a white canvas with the virtual brush (Iosa et al., 2021). In fact, during the task, when painting a virtual masterpiece, the perceived fatigue and the motor errors resulted lower with respect to simply colouring the control canvas (not resembling an art masterpiece): a phenomenon called Michelangelo effect (Iosa et al., 2021). A further study suggested that this effect was stronger for artefacts than for photos of beautiful people, and was modulated by the perceived beauty of the artistic stimulus (Iosa et al., 2022). Then, a virtual art therapy protocol based on this effect was administered to patients with stroke observing an improvement in their neurorehabilitation outcomes (De Giorgi et al., 2023). These results can be explained by recent studies from neuroaesthetics field, suggesting that art may elicit a wide brain arousal also involving motor networks. Some authors have suggested that the activation of motor and premotor areas may be related to the mirror neurons elicited by the observation of the actions performed by the painted figures (Freedberg and Gallese, 2007), or by the recognition of emotions displayed by painted facial expressions (Adolphs et al., 2000). However, these activations were found also when participants observed abstract artworks and it has been suggested that they are related to an empathetic engagement with the implicit imagination of the observer of the movements performed by the artist to create the artworks (Knoblich et al., 2002; Freedberg and Gallese, 2007). So far, the evidence regarding the Michelangelo effect is limited to virtual tasks that require the interaction of 2D stimuli like a painting (Iosa et al., 2021; De Giorgi et al., 2023) or beautiful photos (Iosa et al., 2022). Here, we aims to expand previous evidence by investigate the Michelangelo effect with respect to 3D sculptures. In particular, we realized a motor task in VR for assessing the kinematics and subjective (questionnaire answers) outcomes, where participants used their both hands for sculpting famous sculptures of the history of art, such as the David of Michelangelo, the Venus of Milo and the statue of Laocoon and His Sons and related control stimuli. In this way, we could obtained new evidence for the implementation of a specific virtual task for patients needing bimanual rehabilitation when performing movements in 3D space for favouring neuroplasticity based on sensorimotor learning related to bilateral transfer (Iosa et al., 2013; Fusco et al., 2018; Cacioppo et al., 2023). Moreover, we could also obtain novel insight for implementing a similar task in the Metaverse, allowing patients to perform the motor exercises at home while researcher or medical doctor can virtually monitor the task execution in real time.

We hypothesize that, like virtual painting, virtual sculpting fatigue and performance can be affected by the beauty of the sculpted object, according to the Michelangelo effect. Specifically, we hypothesize that participants performing a virtual sculpting task experience less fatigue and perform more smoothed (Roren et al., 2022) movements when interacting with beautiful artwork, compared to other handicrafts.

## 2. Material and Methods

### 2.1 Participants

Thirty young adults participated to this study (mean age: 25.3 ± 3.7 years; 50% females, students of university courses not related to arts or aesthetics). An Independent Ethical Committee of Santa Lucia Foundation approved the study and all participants signed the informed consent. All the participants declared to have not any cognitive, neurological, orthopaedic or muscular deficit at the moment of the experiment.

### 2.2 Virtual sculpturing task

**Figure 1:**
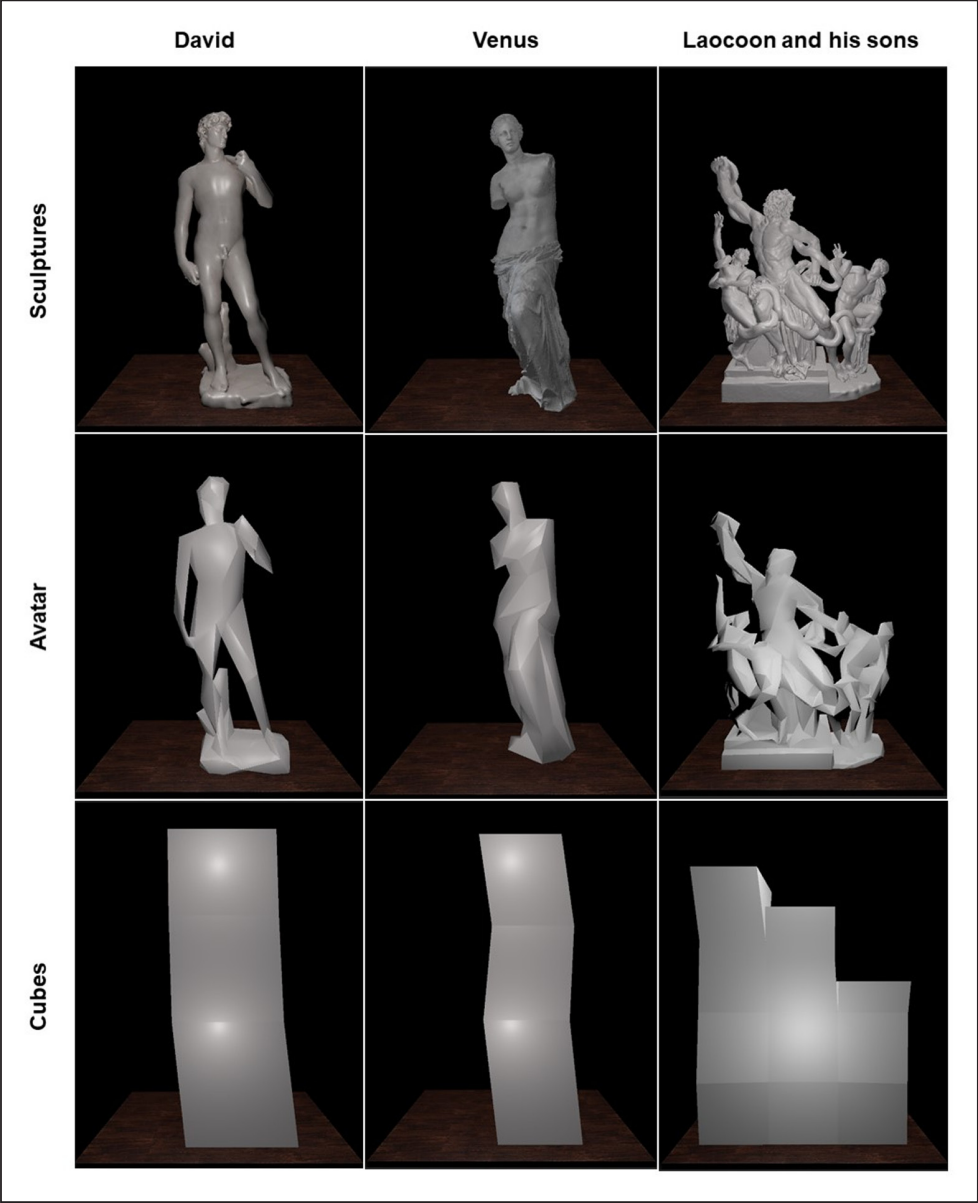
The three types of stimuli: sculptures (digital reproductions of artistic masterpieces, above), avatars (low resolution versions of the sculptures that were reduced to anthropomorphic figures, in the middle), and cubes (geometrical 3D figures formed by cubic voxels covering the same space of relevant sculptures and avatars, below).

Each participant comfortably sat wearing a Meta Quest HMD and holding, in each hand, the Meta Quest joystick, which allowed them to interact with the virtual stimuli. The virtual environment was designed by using Blender 3.1 and implemented in Unity 2018 game engine software, and consisted in a dark room with a spot light that illuminated a wooden pedestal placed in the centre of room. Above the wooden pedestal there was a solid, light grey block, containing a 3D reproduction of famous sculptures from art history or its control stimuli: the David of Michelangelo, the Venus of Milo and the statue of Laocoon and His Sons (Figure 1). In particular, the wooden pedestal was formed by 512 voxels of 5 cm^3^ each (8 length × 16 height × 4 depth) in a volume of 40×80×20 cm for David of Michelangelo and the Venus of Milo stimuli and their control and a block formed by 512 voxels (12 length × 14 height × 4 depth) in a volume of 60×70×20 cm for the statue of Laocoon and His Sons and its controls. We carefully selected these three sculptures from the annals of art history to symbolize distinct human figures: a man, a woman, and a group of people. Moreover, they are emblematic of three distinct concepts: strength, beauty, and suffering, respectively. In our choice of control stimuli, we consciously refrained from utilizing high-resolution digital human figures, as they could be interpreted as digital artworks. We also observed the presence of the Michelangelo effect in beautifully handcrafted works not traditionally regarded as art. Consequently, we opted to employ low-resolution versions of the statues and geometric shapes composed of cuboids, each possessing equivalent volumes to the original sculptures. Similarly, to the previous studies on Michelangelo effect in which a white thin panel covered the already existing painting and the virtual brush deleted the pixels of that panel unveiling the artwork (Iosa et al., 2021), in this task a 3D reproduction of the famous statue was already present inside the block. The participant could see a virtual reproduction of both his/her hands warranting the embodiment, in particular the sense of Agency (Sanchez-Vives et al., 2010), in the digital environment. Each participant was instructed to touch the stone block with their hands to remove parts of the stone and gradually reveal a hidden statue inside. They were also asked to stop when they believed they had completed the task. The voxels touched by the hands disappeared, so that the participant could progressively remove the extra voxels till leaving only the virtual sculpture. Each participant performed 18 trials, in counterbalanced order, in which the above 9 stimuli (3 statue, 3 control low resolution statue and 3 control cubes) were presented twice in a random order. In the following, we referred to these three different types of stimuli as *sculptures* (digital reproductions of artistic masterpieces), *avatars* (low resolution sculptures that were reduced to anthropomorphic figures), and *cubes* (geometrical 3D figures formed by cubic voxels).

### 2.3 Psychometric and kinematic assessment

Trajectories of both hands were recorded in the 3D space by a customized script implemented in Unity and then it was computed the length of these trajectories.

Same kinematic variables assessed in previous studies on the Michelangelo effect were measured, such as the time to complete each trial, the overall percentage of voxels deleted, the percentages deleted by each single hand, and the mean normalized jerk (Iosa et al., 2021, 2022; De Giorgi et al.; 2023). Jerk is a parameter related to the fluidity of movements evaluated along the three axes as the derivate of acceleration integrated with respect to time and normalized for the length and duration of the trial and averaged among the three spatial axes (Iosa et al., 2022). The variable, briefly called symmetry in the following, has been computed as the percentage ratio between the minimum and maximum values recorded between the two hands (so that the value 100% corresponds to the equality of the two values between the two hands).

Similar to previous studies (Iosa et al., 2021, 2022), after each trial, the participants were requested to answer about three questions regarding (1) how much the stimulus was beautiful (objective beauty), (2) how much they liked the stimulus (subjective beauty), and (3) how much tiring was the task (fatigue), by using a numeric rating scale [going from 0 (not at all) to 10 (maximum possible)]. The need to differentiate between objective and subjective beauty may be related to distinct neural circuits involved in these two aesthetic judgments (Di Dio et al., 2007). Furthermore, the participant was also requested to quantify the percentage of ‘how much the right and left hands were used’ (researcher previously instructed the participant that if both the hands were equally used the percentages should be 50% and 50%). These questions were asked to test the correlation between the perception of the participant with respect to the recorded kinematic parameters of his/her performance. At the end of the entire experimental session, the User Satisfaction Evaluation Questionnaire (USEQ) and Nasa Task Load Index (NASA-TLX) were administered to participants. Both these scales test six domains of the self-perception about the usability and perceived load demand of the system. In particular, USEQ has six questions (1. Did you enjoy your experience with the system? 2. Were you successful using the system? 3. Were you able to control the system? 4. Is the information provided by the system clear? 5. Did you feel discomfort during your experience with the system? 6. Do you think that this system will be helpful for rehabilitation?) with a five-point Likert Scale for each one of this item with a score going from 1 to 5, and hence a total score ranging from 6 (poor satisfaction) to 30 (excellent satisfaction). The six items test are related to how much the subject enjoyed the experience, if the subject successfully used the system, if the subject was able to control it, the clarity of the provided information, the perceived discomfort using the system and self-perceived utility about the performed exercise. NASA-TLX has six questions with a ten point numerical rating scale for each one of the item (1. How much mental and perceptual activity was required? 2. How much physical activity was required? 3. How much time pressure did you feel due to the rate or pace at which the tasks occurred? 4. How successful do you think you were in accomplishing the goals of the task set by the experimenter? 5. How hard did you have to work to accomplish your level of performance? 6. How insecure, discouraged, irritated, stressed and annoyed versus secure, gratified, content, relaxed and complacent did you feel during the task?) with a score ranging from 1 to 100. It tests the self-perceived mental demand, physical demand, time demand, effort, performance, and frustration.

### 2.4 Statistical analysis

Data have been reported in terms of mean ± standard deviations. Paired t-test was used in the preliminary analyses. In the following analyses, repeated measure analysis of variance have been performed using as within subject factors the type of the stimuli (sculptures, avatars, cubes), the original model (David, Venus, Laocoon), and also hand laterality for some of the kinematic variables. Post-hoc analyses have been conducted using t-test. Correlations were tested by Pearson’s coefficient. For all the analysis the alpha level of statistical significance was set at 5% with the exception of post-hoc analysis applying the Bonferroni correction to the alpha level.

## 3. Results

### 3.1 Preliminary analysis

A preliminary analysis tested the usability of the virtual reality system by comparing virtual sculpturing with virtual painting, as measured in a previous study (Iosa et al., 2021). Table 1 shows the results of NASA-TLX and USEQ. The frustration measured by NASA-TLX and discomfort measured by USEQ resulted very low. A good level of effort was recorded, together with all the other parameters measured by USEQ. Interestingly, analysing the scores of NASA-TLX, we found that participants perceived the use of the virtual system as more mental demanding (3.81 ± 2.36 on a maximum of 10) than physical demanding (2.56 ± 1.54, p = 0.015, t-test), an opposite results to that found for paintings in the previous study (Iosa et al., 2021).

**Table 1:** Mean ± standard deviation for NASA-TLX (score ranges between 0 and 10) and USEQ (0–5) for sculptures measured in this study and those measured for paintings in a previous study (Iosa et al., 2021).


SCALE	DOMAINS	MEAN ± SD SCULPTURES	MEAN ± SD PAINTINGS

**NASA-TLX**	Mental Demand	3.81 ± 2.36	0.98 ± 1.16

	Physical Demand	2.56 ± 1.54	2.20 ± 2.17

	Temporal Demand	2.19 ± 1.67	0.78 ± 0.11

	Effort	7.88 ± 1.93	1.11 ± 1.06

	Performance	2.94 ± 1.87	2.04 ± 1.99

	Frustration level	1.66 ± 1.54	2.04 ± 1.10

**USE**Q	Experienced enjoyment	4.44 ± 0.67	4.80 ± 0.52

	Successful use	4.69 ± 0.64	4.85 ± 0.37

	Ability to control	4.69 ± 0.59	4.85 ± 0.37

	Clarity of information	4.66 ± 0.87	5.00 ± 0.00

	Discomfort	1.66 ± 1.00	1.65 ± 0.99

	Perceived utility	4.16 ± 1.22	4.70 ± 0.92


### 3.2 Psychometric and Kinematic Analyses

The main hypothesis of this study was that the fatigue and the performance could be affected by the beauty of the sculptured object according to the Michelangelo effect.

**Figure 2:**
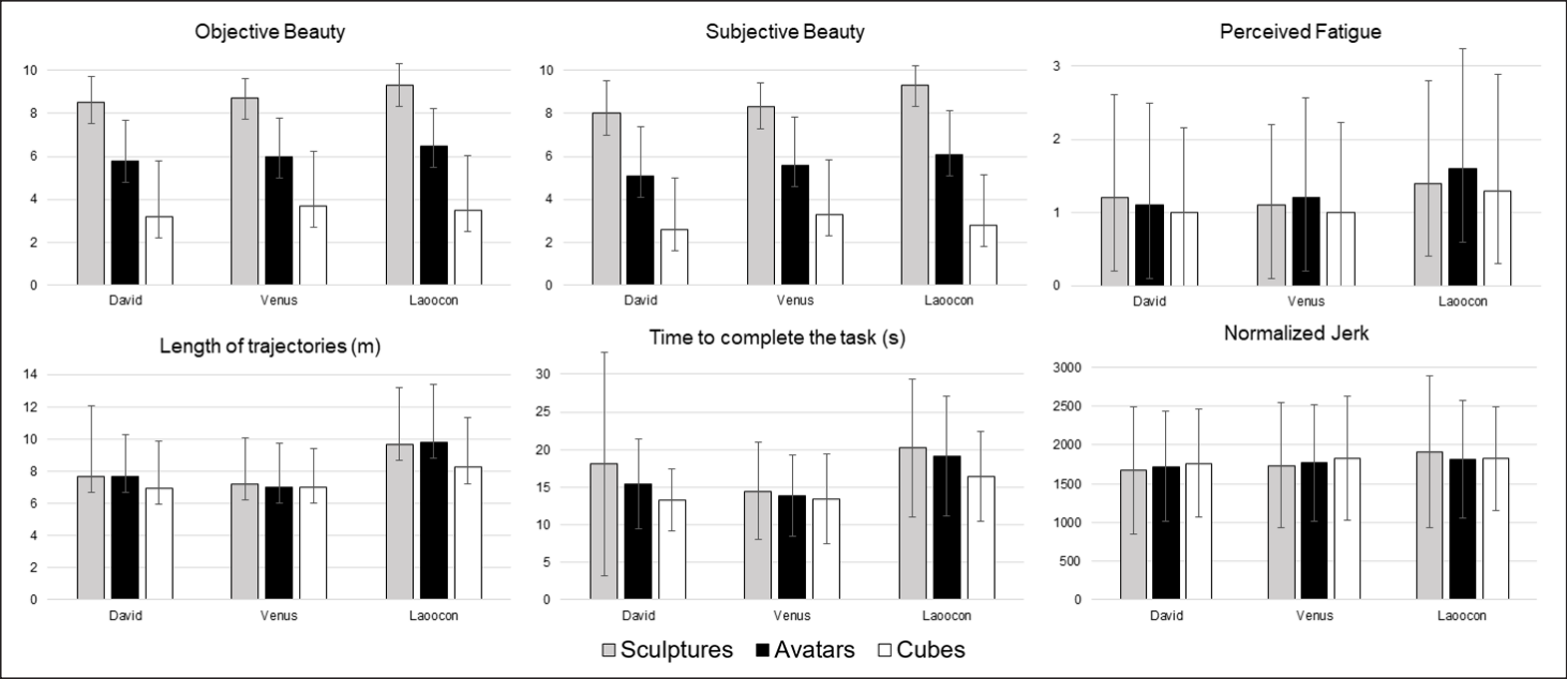
Mean and standard deviation of the kinematic parameters for sculptures (grey bars), avatars (black bars) and cubes (white bars).

As shown in Figure 2, objective and subjective beauty resulted significantly depending on the type of stimuli (p < 0.001, Table 2). Post-hoc analysis showed that the sculptures were preferred to avatars (p < 0.001) and cuboids (p < 0.001), and avatars were preferred to cuboids (p < 0.001), as expected.

**Table 2:** Results of Repeated measure Analysis of Variance on psychometric variables (F and relevant degrees of freedom, p-values in bold if <0.05, and Effect Size ES reported in terms of partial eta squared). Interactions type per model per repetitions were not reported because not statistically significant for all the variables.


PARAMETER	FACTOR	F	P	ES

**Objective Beauty**	Type	F(2,58) = 122.56	**<0.001**	0.809

	Model	F(2,58) = 16.41	**<0.001**	0.361

	Repetition	F(1,29) = 0.80	0.378	0.027

	Type*Model	F(4,116) = 2.56	**0.042**	0.081

	Type*Repetition	F(2,58) = 1.60	0.211	0.052

	Repetition*Model	F(2,58) = 0.49	0.614	0.017

**Subjective Beauty**	Type	F(2,58) = 116.62	**<0.001**	0.801

	Model	F(2,58) = 16.12	**<0.001**	0.357

	Repetition	F(1,29) = 1.91	0.177	0.062

	Type*Model	F(4,116) = 5.96	<0.001	0.170

	Type*Repetition	F(2,58) = 1.16	0.320	0.039

	Repetition*Model	F(2,58) = 1.65	0.201	0.051

**Perceived Fatigue**	Type	F(2,58) = 2.15	0.125	0.069

	Model	F(2,58) = 7.61	**<0.001**	0.274

	Repetition	F(1,29) = 0.36	0.552	0.012

	Type*Model	F(4,116) = 1.80	0.133	0.058

	Type*Repetition	F(2,58) = 3.04	0.056	0.095

	Repetition*Model	F(2,58) = 0.19	0.827	0.007

**Perceived Symmetry**	Type	F(2,58) = 3.17	**0.049**	0.099

	Model	F(2,58) = 7.49	**0.001**	0.205

	Repetition	F(1,29) = 10.28	**0.003**	0.262

	Type*Model	F(4,116) = 0.38	0.820	0.013

	Type*Repetition	F(2,58) = 2.38	0.101	0.076

	Repetition*Model	F(2,58) = 0.38	0.686	0.013


The analysis of kinematic parameters highlighted that the time spent to complete the task depended on the stimulus (p < 0.001, Table 2, Figure 2). This time was longer for sculptures with respect to avatars (post-hoc: p = 0.020) and cuboids (p < 0.001), and for avatars with respect to cuboids (p < 0.001). Similarly, the length of trajectories were not significantly different between sculptures and avatars (post-hoc: p = 0.918), both longer than those recorded for cuboids (p < 0.001). The symmetry evaluated as the ratio between the length of the two hand trajectories depended to the type of stimuli (p = 0.009), as well as the perceived symmetry (p = 0.049), with higher values for cuboids (82 ± 17% and 85 ± 23%, respectively), followed by avatars (78 ± 19% and 84 ± 26%) and sculptures (77 ± 19% and 81 ± 25%). Post-hoc analysis showed that the symmetry of movements was significantly different only between sculptures and cuboids (p < 0.001).

Despite the type of stimulus influenced spatial and temporal parameters, it did not affect the perceived fatigue (p = 0.125). A partial effect was observed related to the interaction type*repetition (p = 0.056).

This interaction was highly significant for the normalized jerk (p < 0.001, Table 3), that was not mainly affected by type of stimuli (p = 0.489). In the first repetition, the mean normalized jerk was 1551 ± 743 for sculptures, 1659 ± 659 for avatars, 1713 ± 659 for cubes whereas in the second repetition: 1993 ± 943, 1878 ± 795, 1896 ± 767, respectively. Post-hoc analysis showed a significant difference in terms of normalized jerk between sculptures and cuboids in the first repetition (p < 0.001) and between sculptures and avatars in the second one (p = 0.019).

**Table 3:** Results of Repeated measure Analysis of Variance on kinematic variables (F and relevant degrees of freedom, p-values in bold if <0.05, and Effect Size ES reported in terms of partial eta squared). Interactions type per model per repetitions were not reported because not statistically significant for all the variables.


**Time to Complete the Task**	Type	F(2,58) = 9.38	**<0.001**	0.319

	Model	F(2,58) = 19.88	**<0.001**	0.499

	Repetition	F(1,29) = 22.74	**<0.001**	0.532

	Type*Model	F(4,116) = 2.08	0.092	0.094

	Type*Repetition	F(2,58) = 1.58	0.219	0.073

	Repetition*Model	F(2,58) = 0.19	0.830	0.009

**Length of Trajectory**	Type	F(2,58) = 11.41	**<0.001**	0.282

	Model	F(2,58) = 68.49	**<0.001**	0.703

	Repetition	F(1,29) = 1.32	0.260	0.044

	Type*Model	F(4,116) = 4.37	**0.002**	0.131

	Type*Repetition	F(2,58) = 0.146	0.864	0.005

	Repetition*Model	F(2,58) = 0.282	0.755	0.010

**Symmetry**	Type	F(2,58) = 5.13	**0.009**	0.150

	Model	F(2,58) = 3.52	**0.036**	0.108

	Repetition	F(1,29) = 6.65	**0.015**	0.186

	Type*Model	F(4,116) = 0.97	0.427	0.032

	Type*Repetition	F(2,58) = 1.45	0.243	0.048

	Repetition*Model	F(2,58) = 0.62	0.539	0.021

**Normalized Jerk**	Type	F(2,58) = 0.72	0.489	0.024

	Model	F(2,58) = 3.60	**0.034**	0.110

	Repetition	F(1,29) = 20.05	**<0.001**	0.409

	Type*Model	F(4,116) = 1.78	0.138	0.058

	Type*Repetition	F(2,58) = 8.82	**<0.001**	0.233

	Repetition*Model	F(2,58) = 011	0.896	0.004


### 3.3 Correlations between perception and action

Table 4 reports the correlations among perception and action related parameters. Some correlations resulted statistically significant for all the types of stimuli: objective with subjective beauty, perceived symmetry with kinematic symmetry, perceived fatigue with time to complete the task and (negatively) with normalized jerk. Other significant correlations were found for a specific type of stimuli. The objective beauty was found correlated with perceived fatigue for cuboids, whereas it was only a trend for sculptures (p = 0.063) and avatars (p = 0.059). Objective beauty was also found correlated with perceived symmetry for avatars. The subjective beauty was found correlated with time to complete the task and normalized jerk only for avatars. Finally, time to complete the task was found significantly correlated with the perceived symmetry for sculptures and avatars.

**Table 4:** Results of correlations between perception and action related parameters (R: Pearson coefficient, *p < 0.05, **p < 0.01, ***p < 0.001).


TYPE OF STIMULUS	VARIABLES	OBJECTIVE BEAUTY	TIME TO COMPLETE THE TASK	NORMALIZED JERK	SYMMETRY

**Sculptures**	**Subjective beauty**	R = 0.627***	R = 0.050	R = 0.100	R = –0.091

	**Perceived fatigue**	R = –0.139	R = 0.286***	R = –0.310***	R = –0.021

	**Perceived symmetry**	R = –0.071	R = –0.164*	R = 0.122	R = 0.727***

**Avatars**	**Subjective beauty**	R = 0.812***	R = –0.185*	R = 0.184*	R = –0.066

	**Perceived fatigue**	R = –0.141	R = 0.311***	R = –0.327***	R = –0.062

	**Perceived symmetry**	R = –0.151*	R = –0.198**	R = 0.122	R = 0.699***

**Cubes**	**Subjective beauty**	R = 0.907***	R = –0.035	R = –0.042	R = –0.001

	**Perceived fatigue**	R = –0.274***	R = 0.247**	R = –0.262***	R = –0.046

	**Perceived symmetry**	R = –0.060	R = –0.130	R = 0.001	R = 0.526***


## Discussion

In the present study, we aimed to expand previous evidence by investigate the Michelangelo effect, observed when painting virtual 2D canvas (Iosa et al., 2021, 2022) with respect to 3D sculptures. In particular, we realized a motor task in VR for assessing the kinematics and subjective (questionnaire answers) outcomes, where participants used their both hands for sculpting famous sculptures from art history and control stimuli. We found high usability ratings for the system. Comparing the scores of USEQ assessed in our protocol of virtual sculpture they were similar not only to those previously assessed for virtual painting (Iosa et al., 2021) as shown in Table 1, but also to those of another virtual system for rehabilitation (Gil-Gomez et al., 2017). In fact, we found similar values with respect to this latter system in terms of experienced enjoyment (4.44 in our study vs. 4.43), successful use (4.69 vs. 4.23), clarity of information (4.66 vs. 4.65), and perceived utility (4.16 vs. 4.00), and better values in terms of ability to control the system (4.69 vs. 3.80) and less discomfort (1.66 vs. 4.70). About NASA-TLX scores, it is noteworthy that the participants judged the task to be more mentally demanding than physically demanding. According to the Michelangelo effect, the main hypothesis of this study was that the parameters related to perception and action were affected by the interaction with the artistic stimuli, in particular about the perceived fatigue and the normalized jerk. First of all, we found some expected results: the sculptures were judged as more beautiful, the fatigue was correlated with the time to complete the task, the perception of a similar use of the two hands was associated to the real difference in the use of the two hands. However, differently from the previous study about virtual painting, in which a significant lower fatigue and shorter trajectories were found in presence of an artistic masterpiece (Iosa et al., 2021), here we did not find a significant main effect of types of handworks on the perceived fatigue for the artistic stimuli. However, it should be observed from Table 4 that, in general, the longer was the time to complete the task the higher was the perceived fatigue, but despite sculptures required longer time (and trajectories) the level of perceived fatigue was not higher than that reported for the other stimuli. So, the reduction of fatigue observed when subjects interacted with artistic paintings in our previous study (Iosa et al., 2021), was not found for sculptures in the present one. This result could be affected by the fact that the time spent to complete the task, a parameter significantly correlated with fatigue, was longer for sculptures than for other stimuli. The perceived fatigue was found related also with the beauty of the stimulus: there was just a trend for sculptures and avatars, but for cubes this correlation resulted statistically significant: lower fatigue were perceived when more pleasant was judged the stimulus. For sculptures this correlation may be affected by a ceiling effect related to the higher scores given in terms of beauty. These results are similar to those of a recent electroencephalographic study that showed high positive emotional responses associated to visual art stimuli, regardless of their aesthetics, and to commercial stimuli only when they were regarded as beautiful (Cheung et al., 2019). The jerk, i.e. the parameter related to the smoothness of the movements, was found significantly lower for sculptures, but only at the first repetition of each stimuli. A recent study highlighted the need of a correct match between skill and difficulty of the task in VR to led to higher levels of flow, to avoid a reduction of the quality of the performance, physiological arousal, and enjoyment (Lemmens and von Münchhausen, 2023). In our experiment, the replication of sculptures may have reduced the engagement of participants. For the first repetition, the results were in line with the Michelangelo effect that already showed a more smoothed hand trajectories (lower jerk) for beautiful than for non-beautiful stimuli (Iosa et al., 2022). The smoothness is an important characteristics of fluid physiological hand movements that inspired to Luria the concept of kinetic melodies (Luria & Hutton, 1977). For example, the harmony of body proportions also was found replicated in the harmony of human walking, both found in golden ratio. This number was also recently found in the Michelangelo masterpiece the Creation of Adam, at the centre of Sistine Chapel, as the horizontal proportion between the representation of God and that of Adam (de Campos et al., 2015). This ratio was also investigated in many studies covering more than 150 years of psychological studies about aesthetics (Fechner, 1865; De Bartolo et al., 2022).

Our data interpretation need cautions for taking into the limits of our study and also the fact that correlations, despite statistically significant, had not so high values of Pearson’s coefficient. Then, we tested only three models (David, Venus and Laocoon) of artistic sculptures. Although none of our participants were studying arts or aesthetics in university, another limitation of our study was that we did not assess their artistic knowledge or inclination.

The general finding of our study seems to confirm that the aesthetic experience may affect the perception and the action of participants. Neuroaesthetics has been defined as a relatively new (sub)field which aims to understand the brain mechanisms that are engaged during aesthetic and allied experiences in the widest sense (Zeki et al., 2020). Our results should be read with a translational approach: they could be helpful in understanding and improving the efficacy of art therapy protocols. The scientific findings reported in the papers of neuroaesthetics are often poorly considered for designing art therapy protocols, despite their potential to improve the efficacy of the interventions (Oliva et al., 2023). Conversely, the neuroaesthetic findings related to the Michelangelo effect (Iosa et al., 2021) were at the basis of an art therapy protocol that improved the outcomes of neurorehabilitation in patients with stroke (De Giorgi et al., 2023). However, the present study showed a less pronounced Michelangelo effect in virtual sculpturing compared to that previously observed in virtual painting. Several factors may have contributed to the lack of a statistically significant effect on perceived fatigue: the task was performed bimanually, it was perceived as more mentally demanding, and notably, the time required to complete the task was longer for statues compared to other stimuli. Regarding the performed movements, they resulted smoother for artistic sculptures, but only for the first presentation of each statue. The present study revealed that artistic stimuli were associated with smoother movements in virtual sculpturing (though not when a trial already done was presented again) and were not perceived as more tiring, despite the longer time required for completion compared to other stimuli. Further studies carried out on clinical populations should investigate whether virtual sculpting could be an effective protocol of art therapy, especially for patients requiring bilateral rehabilitation (Norouzi et al., 2021) or for patients, such as those with stroke, who may benefit of therapy protocols based on the bilateral transfer (Iosa et al., 2013).

## Prospective for motor neurorehabilitation in the Metaverse

We can speculate regarding three possible applications of this evidence regarding the art therapy and neuroaesthetics principles for motor rehabilitation in the Metaverse with VR.

First, the realization of an immersive virtual environment, composed by different art masterpieces, like paintings and sculptures, where participants can actively interact with them (by using their real hands), could increase the well-being in the observers and can act on different brain mechanisms. It may involve mirror neuron networks (activated by the actions performed by represented persons; Freedberg and Gallese, 2007), the motor imagery related to possibility of using painted objects or of exploring the scene (Di Dio et al., 2016), the recognition of emotions displayed by expressions of painted persons (Adolphs et al., 2000), and even the empathetic engagement with the movements performed by the artist to create the artworks (Knoblich et al., 2002; Freedberg and Gallese, 2007). Thus, the exposure to an artistic virtual environment can boost the efficacy of motor rehabilitation (De Giorgi et al. 2023) by increasing the patients’ motivation and interest, the intensity and the amount of time for daily treatment, which represent an important step forward for improving the rehabilitative applications (Fusco & Tieri 2022).

Second, it is important to point out the role of virtual embodiment (Kilteni et al. 2012), i.e. the illusion to own a virtual body (ownership) and have the control over its actions (agency), for further applications of art therapy in VR and, in general, in the Metaverse. Previous evidence showed that this kind of illusion can be induced by observing the virtual body from a first person perspective point of view (Tieri et al. 2015a, 2017) and can be further enhanced when the virtual body synchronously moves with the observer’s real movements (Sanchez-Vives et al. 2010), or when the virtual body is touched while the observer simultaneously feels the touch on corresponding real body areas (Slater et a. 2008). It is worth noting that virtual body illusion acts at both explicit, i.e. questionnaire answers (Tieri et al. 2015a; Fusco et al. 2021), and implicit level, i.e. physiological (Tieri et al. 2015b, 2017) and neural activity (Casula et al. 2022; Spinelli et al. 2018), and can also affect the perceptions, attitudes and behaviours (Yee & Bailenson, 2007). Indeed, recent results shows that embodying virtual bodies resembling certain specific individuals, e.g. Einstein (Banakou et al. 2018), Freud (Osimo et al. 2015), Michelangelo’s representation of God (Frisanco et al. 2022), leads people to adhere to the new identities by behaving accordingly with the hallmarks of the characters they were embodying. In this line, we can argue that inducing virtual embodiment, for example the body of a great artist of the history of art like Michelangelo, can improve the efficacy of the VR art therapy. It should be noted that, in the present study, we included only virtual hands that were synchronously moved by the participant’s real hands, but not the full virtual body, in order to avoid any additional effects due to the virtual embodiment. However, future studies are necessary to better understand how the virtual embodiment can be exploited for improving the efficacy of VR rehabilitative applications. Finally, considering the Metaverse as a social virtual environment, we can imagine a possible VR application, where neurologic patients (after a training in laboratory or hospital) can perform the art therapy at home, while the researcher, physiotherapist or medical doctor, physically dislocated at hospital (tele-presence), can monitor the execution of motor exercises in real-time (Perez-Marcos et al. 2012; Riva et al., 2021). This kind of application can be useful for the reduction of time and costs for reaching the hospital and for allowing a single medical doctor or physiotherapist to monitor more patients at the same time, without occupying real spaces in hospital.

## Data Accessibility Statement

The data are available via the Open Science Framework: https://osf.io/m7zau/.
